# *In Utero* DDT and DDE Exposure and Obesity Status of 7-Year-Old Mexican-American Children in the CHAMACOS Cohort

**DOI:** 10.1289/ehp.1205656

**Published:** 2013-03-19

**Authors:** Marcella Warner, Raul Aguilar Schall, Kim G. Harley, Asa Bradman, Dana Barr, Brenda Eskenazi

**Affiliations:** 1 Center for Environmental Research and Children’s Health, School of Public Health, University of California, Berkeley, Berkeley, California, USA; 2 Division of Laboratory Sciences, National Center for Environmental Health, Centers for Disease Control and Prevention, Atlanta, Georgia, USA; 3 Emory University, Rollins School of Public Health, Atlanta, Georgia, USA

**Keywords:** body mass index, children, dichlorodiphenyltrichloroethane, dichlorodiphenyldichloroethylene, obesity, prenatal exposure

## Abstract

Background: *In utero* exposure to endocrine disrupting compounds including dichlorodiphenyltrichloroethane (DDT) and dichlorodiphenyldichloroethylene (DDE) has been hypothesized to increase risk of obesity later in life.

Objectives: The Center for the Health Assessment of Mothers and Children of Salinas (CHAMACOS) study is a longitudinal birth cohort of low-income Latinas living in a California agricultural community. We examined the relation of *in utero* DDT and DDE exposure to child obesity at 7 years of age. We also examined the trend with age (2, 3.5, 5, and 7 years) in the exposure–obesity relation.

Methods: We included 270 children with *o,p´*-DDT, *p,p´*-DDT, and *p,p´*-DDE concentrations measured in maternal serum during pregnancy (nanograms per gram lipid) and complete 7-year follow-up data including weight (kilograms) and height (centimeters). Body mass index (BMI; kilograms per meter squared) was calculated and obesity was defined as ≥ 95th percentile on the sex-specific BMI-for-age Centers for Disease Control and Prevention 2000 growth charts.

Results: At 7 years, 96 (35.6%) children were obese. A 10-fold increase in *o,p´*-DDT, *p,p´*-DDT, or *p,p´*-DDE, was nonsignificantly associated with increased odds (OR) of obesity [*o,p´*-DDT adjusted (adj-) OR = 1.17, 95% CI: 0.75, 1.82; *p,p´*-DDT adj-OR = 1.19, 95% CI: 0.81, 1.74; *p,p´*-DDE adj-OR = 1.22, 95% CI: 0.72, 2.06]. With increasing age at follow-up, we observed a significant trend toward a positive association between DDT and DDE exposure and odds of obesity.

Conclusion: We did not find a significant positive relation between *in utero* DDT and DDE exposure and obesity status of 7-year-old children. However, given the observed trend with age, continued follow-up will be informative.

*In utero* exposure to endocrine-disrupting compounds has been hypothesized to increase risk of obesity in childhood and into adulthood (Baillie-Hamilton 2002; Diamanti-Kandarakis et al. 2009; Heindel and vom Saal 2009). Increasing animal evidence supports a potential role of endocrine-disrupting compounds either directly or indirectly in the pathogenesis of obesity (Grun and Blumberg 2006; Heindel and vom Saal 2009; Newbold et al. 2007, 2009). Early-life exposure might alter development of adipose tissue in terms of number, size, and distribution of adipocytes formed, or it may affect the larger regulatory systems involved in weight homeostasis (Grun and Blumberg 2009).

The compound, dichlorodiphenyltrichloroethane (DDT), and its primary metabolite, dichlorodiphenyldichloroethylene (DDE), are persistent organic pollutants and known endocrine disruptors (Agency for Toxic Substances and Disease Registry 2002). A wide range of reproductive and developmental effects have been associated with DDT and DDE exposure (Eskenazi et al. 2009), but recent studies have focused on possible obesogenic effects of these compounds. *In vitro* studies in two preadipocyte cell lines (3T3-L1, 3T3-F442) suggest that *p,p*´-DDT has the ability to alter adipocyte differentiation, and that these effects correlate with changes induced in the expression of C/EBPα (CCAAT/enhancer-binding protein α) and PPARγ (peroxisome proliferator-activated receptor γ), the main transcription factors regulating the adipogenic process (Moreno-Aliaga and Matsumura 2002). The mechanisms leading to the stimulation of these two nuclear factors, however, remain unclear. In a similar *in vitro* study, *p,p*´-DDE had no effect on adipogenesis, but was shown to promote fatty acid uptake under nonstimulated conditions in mature adipocytes (Howell and Mangum 2011). Thus, experimental evidence suggests that both compounds have the ability to promote some aspects of adipose dysfunction.

Results of epidemiologic studies of prenatal DDT and DDE exposure and child growth are inconsistent and direct comparison is limited by variations in exposure and outcome assessment (Burns et al. 2012; Cupul-Uicab et al. 2010; Gladen et al. 2000, 2004; Jusko et al. 2006; Karmaus et al. 2009; Mendez et al. 2011; Pan et al. 2010; Ribas-Fito et al. 2006; Valvi et al. 2012; Verhulst et al. 2009). To date, three studies have utilized both a direct measure of *in utero* exposure in maternal serum during pregnancy or cord blood and an age-standardized measure of overweight based on body mass index (BMI) *z*-score ≥ 85th percentile (Mendez et al. 2011; Valvi et al. 2012; Verhulst et al. 2009). All three studies reported positive associations between prenatal DDE exposure and overweight status with follow-up periods ranging from 14 months to 6.5 years, but only one study reported the association with DDT exposure. In a prospective birth cohort of 518 children in Spain, maternal serum DDE was associated with increased risk of overweight (BMI *z*-score ≥ 85th percentile) at 14 months of age (Mendez et al. 2011). DDT was measured but excluded from analysis due to the low detection frequency (99% nondetect). Additionally, higher cord blood DDE levels (DDT was not measured) were associated with increased BMI SD score at 3 years of age in a prospective birth cohort study of 138 children in Belgium (Verhulst et al. 2009). The association was enhanced among children of smoking mothers. Finally, in a prospective birth cohort of 344 children in Spain, higher cord blood *p,p*´*-*DDT and *p,p*´*-*DDE levels were nonmonotonically associated with increased BMI *z*-score and odds of overweight (≥ 85th percentile) at 6.5 years, but associations were modified by child sex (Valvi et al. 2012). The association with *p,p*´*-*DDT was limited to males, and the association with *p,p*´*-*DDE was stronger among females.

In the present study, we examined the relation of maternal serum concentrations of *o,p*´*-*DDT, *p,p*´*-*DDT, and *p,p*´*-*DDE (DDT and DDE) during pregnancy with obesity status of 7-year-old children in the Center for the Health Assessment of Mothers and Children of Salinas (CHAMACOS) study, a longitudinal birth cohort study in a California agricultural community. We hypothesized that prenatal exposure to DDT and DDE would alter risk for obesity later in life.

## Methods

*Study participants*. The CHAMACOS study is a longitudinal birth cohort study of the effects of pesticide and other environmental exposures on the health of pregnant women and their children living in the Salinas Valley, an agricultural region in California. Pregnant women were recruited between October 1999 and October 2000. Eligible women were ≥ 18 years of age, < 20 weeks gestation at enrollment, English- or Spanish-speaking, qualified for government-sponsored health care, and planned to deliver at the county hospital. The study was approved by the institutional review boards at participating institutions and written informed consent was obtained from all mothers.

Of 601 women who were initially enrolled, 527 were followed through delivery of a singleton live birth that survived the neonatal period, and 417 provided a maternal serum sample during pregnancy for DDT and DDE analysis. Of these, complete follow-up interview and anthropometric measurements were available for children at 2 years (*n* = 307), 3.5 years (*n* = 271), 5 years (*n* = 265), and 7 years (*n* = 270) of age. Thus, the main study sample included the 270 children with 7-year data.

*Procedure.* Details of the study are presented elsewhere (Eskenazi et al. 2003). Briefly, after informed consent was obtained, women were interviewed in English or Spanish by trained, bilingual, bicultural interviewers twice during pregnancy [first: mean, 13 weeks (range, 4–29), second: mean, 26 weeks (range, 18–39) gestation], shortly after delivery, and when their children were 6 months and 1, 2, 3.5, 5, and 7 years of age. During each interview, we collected information about family sociodemographic characteristics, household member work histories, maternal characteristics and personal habits, pregnancy and medical histories, and child-based developmental milestones, diet, and behavioral information.

We made child anthropometric measurements including weight (kilograms) and height (centimeters) at each follow-up visit. Beginning at 2 years of age, we measured barefoot standing height to the nearest 0.1 cm using a stadiometer and standing weight to the nearest 0.1 kg using a digital scale (Tanita Mother-Baby scale, model 1582; Tanita Corp., Arlington Heights, IL). Starting at age 5 years, we also measured waist circumference to the nearest 0.1 cm by placing a measuring tape around the abdomen at the level of the iliac crest, parallel to the floor. All measurements were made in triplicate and averaged for analysis.

*Laboratory analyses*. Maternal serum samples were collected by venipuncture during routine glucose tolerance testing at approximately 26 weeks gestation. Serum levels of *o,p*´*-*DDT, *p,p*´*-*DDT, and *p,p*´*-*DDE were measured by isotope dilution gas chromatography–high resolution mass spectrometry methods (Barr et al. 2003), and reported on a whole-weight basis (picograms per gram). The sample mean (± SD) levels of detection for *o,p*´*-*DDT, *p,p*´*-*DDT, and *p,p*´*-*DDE were 1.2 ± 0.6, 1.5 ± 0.8, and 2.9 ± 1.4 pg/g serum, respectively. For nondetectable values, a serum level equal to one-half the detection limit was assigned (Hornung and Reed 1990). Lipid-adjusted values (nanograms per gram) were calculated by dividing *o,p*´*-*DDT, *p,p*´*-*DDT, and *p,p*´*-*DDE on a whole-weight basis by total serum lipid content, estimated by enzymatic determination of triglycerides and total cholesterol (Phillips et al. 1989).

*Statistical analyses*. Lipid-adjusted levels of *o,p*´-DDT, *p,p*´*-*DDT, and *p,p*´*-*DDE were log_10_-transformed and analyzed as continuous variables. We calculated BMI at 2, 3.5, 5, and 7 years as weight (kilograms) divided by height (meters) squared. We calculated age- and sex-specific BMI *z*-scores and percentiles for each child using 2000 Centers for Disease Control and Prevention growth charts (Kuczmarski et al. 2002). Obesity was defined as being at or above the 95th percentile of the sex-specific BMI for each child’s age. Overweight was defined as being at or above the 85th percentile, but less than the 95th percentile of sex-specific BMI for age. Normal weight was defined as being less than the 85th percentile of sex-specific BMI for age. Waist circumference was dichotomized into at or below versus above the 90th percentile of sex-specific waist circumference for age.

All statistical analyses were performed using Stata 11.2 (StataCorp, College Station, TX). We used linear regression to examine the relation of log_10_-transformed maternal serum DDT and DDE concentrations with continuous outcomes (BMI *z*-score). We used logistic regression to examine the relation of log_10_-transformed maternal serum DDT and DDE concentrations with categorical outcomes including obesity (≥ 95th vs. < 95th percentile), overweight or obesity (≥ 85th vs. < 85th percentile), and waist circumference (≥ 90th vs. < 90th percentile). We used polytomous logistic regression to examine the relation of log_10_-transformed maternal serum DDT and DDE concentrations with ordered three-category weight outcome [obese (≥ 95th percentile), overweight (< 95th and ≥ 85th percentile), normal weight (< 85th percentile)]. For all regression models, standard errors were estimated using the robust Huber–White sandwich estimator. We performed regression diagnostics to ensure no unduly influential data points with standardized residuals > 3 or < –3, but there were none.

We examined the effect of potential confounding variables identified *a priori* in the child obesity literature (Ebbeling et al. 2002). Potential confounders considered included family socioeconomic status (≤ poverty level, > poverty level), language spoken in the home (mostly Spanish, English/Spanish equally, mostly English), maternal education level (≤ 6th grade, 7–12th grade, ≥ high school), maternal marital status (not married vs. married/living as married), number of years mother had lived in the United States at the time of pregnancy (≤ 1, 2–5, 6–10, ≥ 11), maternal country of birth (United States, Mexico, other), maternal age (years), maternal prepregnancy BMI (categorical) (from reported weight and measured height at initial interview), maternal smoking during pregnancy (no, yes), maternal soda consumption during pregnancy (sodas/week), maternal BMI when the child was 7 years of age (measured weight and height), child sex, child birth weight (continuous), child birth order, whether child was breastfed [no, yes (duration in months)], child age at follow-up (months), and child health behaviors including intake of diet and regular soda, sweetened beverages, fast food, and sweet snacks, time spent watching television (hours/day), and time spent playing outside (hours/day) (maternal report). Covariates were kept in the model if they changed the coefficient for exposure (log_10_ DDT/DDE) by > 10% or if they were independently associated with the outcome at *p* < 0.10. Final covariates included maternal prepregnancy BMI, birth weight, and age-specific time spent watching television (continuous hours/day). We also considered possible interaction of child sex (male vs. female), breastfeeding status (continuous in months), and maternal prepregnancy BMI (continuous) with the exposure in all analyses by including a product term between exposure and effect modifier. Interactions were considered significant if the *p-*value for the interaction term was < 0.2.

Using all children with any growth data at ages 2, 3.5, 5, or 7 years (*n* = 334, average number of observations = 3.3), we examined the trend with age at follow-up for BMI *z*-score and odds of obesity. We used a generalized estimating equation (GEE) model with the same set of covariates included in the 7-year models and an additional interaction term between exposure and exact age of child at time of evaluation. We considered the age interaction to be significant if the interaction term *p*-value was < 0.20. We used the Stata *lincom* postestimation command to calculate the mean beta coefficient for BMI *z*-score (95% CI) or odds ratio (OR) for obesity (95% CI), respectively, at each of the ages of interest. In sensitivity analyses, we repeated the final models excluding children who were low birth weight (*n* = 9) or preterm (*n* = 20). We also repeated the GEE models, limiting the analysis to children (*n* = 230) with complete data at all four follow-up periods.

## Results

Table 1 presents maternal and child characteristics of the CHAMACOS birth cohort by obesity status at 7 years of age. Most mothers were Latina (98%), Mexican-born (89.6%), had not completed high school (78.5%), and were living at or below the federal poverty line (70.0%). At the time of the pregnancy, mothers were an average (± SD) of 26.1 ± 5.0 years old. Almost all mothers (96%) initiated breastfeeding and the mean length of breastfeeding was 9.0 ± 8.3 months. Before pregnancy, 64.1% of mothers were overweight or obese (mean BMI = 27.6 ± 5.5 kg/m^2^) and, by the 7-year follow-up, this number had increased to 86.3% (mean BMI = 31.4 ± 6.2 kg/m^2^).

**Table 1:** Maternal and child characteristics by child obesity status at 7 years of age, CHAMACOS, 2007–2008.

Characteristic	n (%)	Obesea n (%)	Not obese n (%)
Total	270 (100.0)	96 (35.6)	174 (64.4)
Maternal characteristics
Country of birth
USA	28 (10.4)	11 (11.5)	17 (9.8)
Mexico/other	242 (89.6)	85 (88.5)	157 (90.2)
Race/ethnicity
Caucasian	2 (0.7)	2 (2.1)	0 (0.0)
Latina	265 (98.2)	94 (97.9)	171 (98.3)
Other	3 (1.1)	0 (0.0)	3 (1.7)
Years of residence in USA
≤ 5	133 (49.3)	43 (44.8)	90 (51.7)
> 5	137 (50.7)	53 (55.2)	84 (48.3)
Education
≤ 6th grade	120 (44.4)	44 (45.8)	76 (43.7)
7th–12th grade	92 (34.1)	33 (34.4)	59 (33.9)
≥ High school	58 (21.5)	19 (19.8)	39 (22.4)
Marital status
Not married	14 (5.2)	5 (5.2)	9 (5.2)
Married/living as married 256 (94.8) 91 (94.8) 165 (94.8)
Socioeconomic status
At or below poverty	189 (70.0)	66 (68.8)	123 (70.7)
Above poverty	81 (30.0)	30 (31.2)	51 (29.3)
Prepregnancy BMI*
Underweight	2 (0.7)	0 (0.0)	2 (1.2)
Normal	95 (35.2)	25 (26.0)	70 (40.2)
Overweight	105 (38.9)	33 (34.4)	72 (41.4)
Obese	68 (25.2)	38 (39.6)	30 (17.2)
Smoke during pregnancy
No	259 (95.9)	90 (93.8)	169 (97.1)
Yes	11 (4.1)	6 (6.2)	5 (2.9)
Soda during pregnancyb
< 1 per week	134 (50.2)	39 (41.0)	95 (55.2)
1–6 per week	103 (38.6)	43 (45.3)	60 (34.9)
≥ 1 per day	30 (11.2)	13 (13.7)	17 (9.9)
Age at delivery (years)
< 25	111 (41.1)	39 (40.6)	72 (41.4)
25–34	137 (50.7)	47 (49.0)	90 (51.7)
34	22 (8.2)	10 (10.4)	12 (6.9)
Breastfeeding duration (months)
0–2	68 (25.2)	22 (22.9)	46 (26.4)
2–6	66 (24.4)	24 (25.0)	42 (24.1)
6–12	63 (23.3)	18 (18.8)	45 (25.9)
> 12	73 (27.0)	32 (33.3)	41 (23.6)
Table 1. Maternal and child characteristics by child obesity status at 7 years of age, CHAMACOS, 2007–2008.

The 270 children were an average of 7.1 ± 0.2 years old at the 7-year follow-up and 53.7% were female (Table 1). At birth, the children weighed an average of 3,462 ± 499 g, 9 (3.3%) were low birth weight (< 2,500 g), 20 (7.4%) were preterm (< 37 weeks), and about one-third were first-born. The 7-year-old children watched television an average of 2.0 ± 1.1 hr/day and played outside an average of 2.0 ± 1.4 hr/day. About half (53%) of children consumed less than one soda per week, but 40% consumed one or more sodas per week, and 7% consumed one or more sodas per day.

At the 7-year follow-up, the mean (± SD) BMI *z*-score for the 270 children was 1.12 ± 1.0. In total, 96 (35.6%) children were classified as obese and an additional 48 (17.8%) were overweight. A total of 91 (33.7%) children had a waist circumference ≥ 90th percentile for age, and of these, 82 (90.1%) were also obese. As presented in Table 1, obese children were more likely to have an obese mother prepregnancy (*p* < 0.001) and at the 7-year follow-up (*p* < 0.001). Obese children were also more likely to have higher birth weight (*p* = 0.05), and watch television > 2 hr/day (*p* = 0.01). There was no significant difference in obesity status of children by child dietary factors including soda consumption or maternal sociodemographic indicators, including mother’s country of birth, years lived in the United States, education, poverty, or marital status. The association with covariates was similar when different obesity measures (overweight, three-category weight, BMI *z*-score, waist circumference) were considered (data not shown).

Maternal serum levels of *o,p*´*-*DDT, *p,p*´*-*DDT, and *p,p*´*-*DDE were above the limit of detection for 100%, 96%, and 100% of the samples, respectively. The geometric mean [± geometric SD (GSD)] serum levels were 1.66 (± 4.2) ng/g lipid *o,p*´*-*DDT, 20.45 (± 5.1) ng/g lipid *p,p*´*-*DDT, and 1,422 (± 3.3) ng/g lipid *p,p*´*-*DDE. Similar to what has been reported previously (Bradman et al. 2007), maternal levels of *o,p*´-DDT, *p,p*´-DDT, and *p,p*´-DDE were significantly (*p* = 0.05) higher among mothers who were Mexican-born, had lived in the United States ≤ 5 years, and were less educated (≤ 6th grade) (data not shown). Maternal levels were also significantly positively associated with longer duration of breastfeeding, but not with maternal BMI (prepregnancy or at child’s 7 years), child age, sex, birth order, or birth weight. Maternal levels, however, were significantly negatively associated with time the child spent watching television (*p* < 0.05) at 7 years.

As presented in Table 2, maternal serum concentrations in pregnancy of DDT and DDE were nonsignificantly positively related to odds of obesity and overweight when the child was 7 years of age, but not increased waist circumference. After adjusting for maternal prepregnancy BMI, birth weight, and child television time, a 10-fold increase in *o,p*´-DDT or *p,p*´-DDT was nonsignificantly associated with increased odds of obesity [*o,p*´-DDT adjusted (adj-) OR = 1.17; 95% CI: 0.75, 1.82; *p,p*´-DDT adj-OR = 1.19; 95% CI: 0.81, 1.74] and overweight (*o,p*´-DDT adj-OR = 1.32; 95% CI: 0.87, 2.00; *p,p*´-DDT adj-OR = 1.26; 95% CI: 0.87, 1.83). Results for DDE exposure were similar. A 10-fold increase in *p,p*´-DDE, was nonsignificantly associated with increased odds of obesity (adj-OR = 1.22; 95% CI: 0.72, 2.06) and overweight (adj-OR = 1.40, 95% CI: 0.84, 2.33). When we considered obesity status as an ordered three-category variable (obese, overweight, normal weight), the results for DDT and DDE were consistent with estimates for the dichotomous outcomes (data not shown). We found no evidence of an association between DDT and DDE exposure and odds of waist circumference ≥ 90th percentile, after adjusting for maternal prepregnancy BMI, birth weight, and child television time (Table 2).

**Table 2:** Results of logistic regression models for associations of *in utero* DDT and DDE exposure with childhood obesity, overweight or obesity, and waist circumference at 7 years, CHAMACOS, 2007–2008.

Outcome	Cases (%)/total	Exposure	Crude OR (95% CI)	Adjusteda OR (95% CI)
Obesity	96 (35.6)/270	log o,p’-DDT	1.08 (0.73, 1.61)	1.17 (0.75, 1.82)
		log p,p’-DDT	1.04 (0.73, 1.47)	1.19 (0.81, 1.74)
		log p,p’-DDE	1.05 (0.65, 1.70)	1.22 (0.72, 2.06)
Overweight or obesity	144 (53.3)/270	log o,p’-DDT	1.32 (0.89, 1.96)	1.32 (0.87, 2.00)
		log p,p’-DDT	1.19 (0.85, 1.68)	1.26 (0.87, 1.83)
		log p,p’-DDE	1.27 (0.80, 2.02)	1.40 (0.84, 2.33)
Increased waist circumference	91 (33.7)/270	log o,p’-DDT	1.00 (0.66, 1.50)	1.00 (0.63, 1.56)
		log p,p’-DDT	0.92 (0.64, 1.32)	0.97 (0.65, 1.44)
		log p,p’-DDE	0.91 (0.56, 1.48)	0.95 (0.56, 1.62)
aAdjusted for maternal prepregnancy BMI, child television time, and birth weight.				

**Figure 1:**
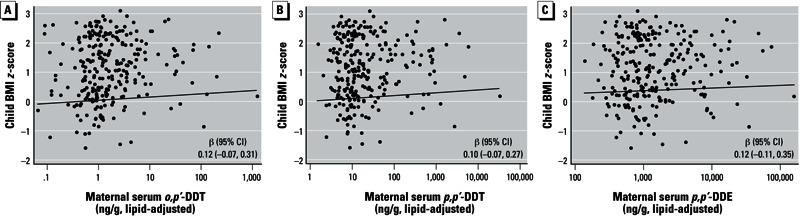
Linear regression models of child BMI z-score at 7 years with *in utero* exposure to *o,p´*‑DDT (*A*), *p,p´*‑DDT (*B*), and *p,p´*‑DDE (*C*), CHAMACOS, 2007–2008. Models were adjusted for maternal prepregnancy BMI, child television time, and birth weight.

Figure 1 presents the relationship of *in utero* DDT and DDE exposure with BMI *z*-score. After adjusting for maternal prepregnancy BMI, birth weight, and child television time, a 10-fold increase in *o,p*´-DDT or *p,p*´-DDT was nonsignificantly positively associated with BMI *z*-score (*o,p*´-DDT adj-β = 0.12; 95% CI: –0.07, 0.31; *p,p*´-DDT adj-β = 0.10; 95% CI: –0.07, 0.27). *In utero* exposure to *p,p*´-DDE was similarly nonsignificantly positively associated with BMI *z*-score (*p,p*´-DDE adj-β = 0.12; 95% CI: –0.11, 0.35).

We found no evidence of effect modification for any of the models (*p*-interaction > 0.20) by child sex, breastfeeding status, or maternal prepregnancy BMI (data not shown). We repeated the final models excluding 20 children who were preterm delivery or 9 who were low birth weight, and the results were not different (data not shown). The children included in the analysis did not differ significantly from those who were excluded due to missing prenatal exposure or 7-year anthropometric data in terms of maternal characteristics (education, marital status, income), maternal prepregnancy BMI or child birth weight, maternal serum DDT and DDE levels, or child obesity status (data not shown).

**Figure 2:**
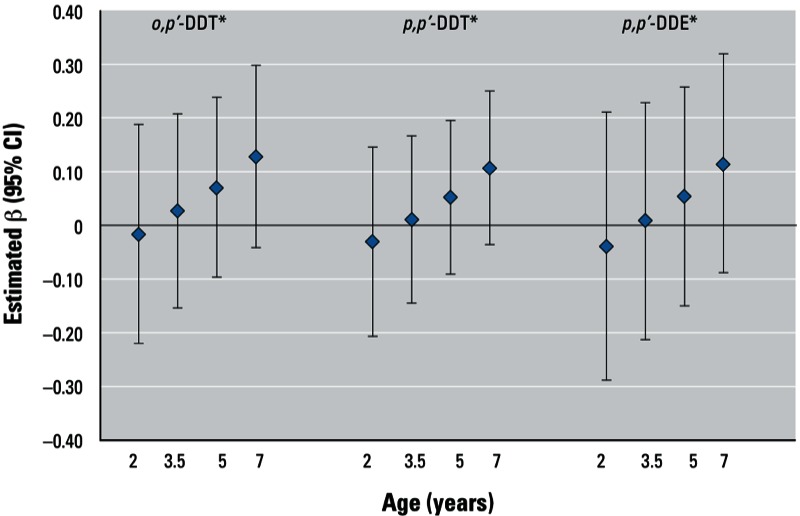
Trend in estimated association for BMI z-score at 2, 3.5, 5, and 7 years with *in utero* log DDT and DDE exposure, CHAMACOS 2007–2008. Age-specific association was derived using lincom after GEE model for exposure and interaction with exact age at measurement. Measures were based on participants with growth data at any age (*n* = 334, average no. of observations = 3.3). All models were adjusted for child’s exact age in months, maternal prepregnancy BMI, birth weight, and child age-specific television time. *p-interaction = 0.123 for *o,p’*-DDT; *p*-interaction = 0.087 for *p,p’*-DDT; *p*-interaction = 0.196 for *p,p’*-DDE.

**Figure 3:**
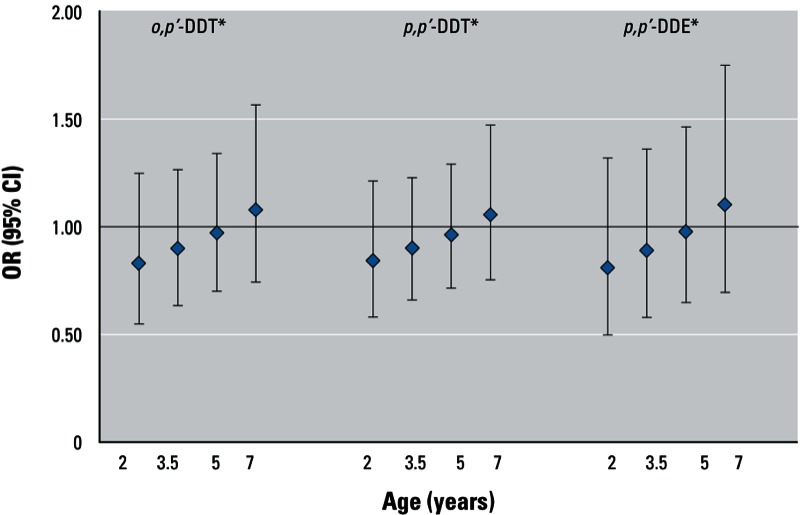
Trend in estimated OR for obesity at 2, 3.5, 5, and 7 years with *in utero* log DDT and DDE exposure, CHAMACOS 2007–2008. Age-specific associations were derived using lincom after GEE model for exposure and interaction with exact age at measurement. Measures were based on participants with growth data at any age (*n* = 334, average no. of observations = 3.3). All models were adjusted for child’s exact age in months, prepregnancy BMI, birth weight, and child age-specific television time. *p-interaction = 0.192 for *o,p’*‑DDT; *p*-interaction = 0.215 for *p,p’*-DDT; *p*-interaction = 0.185 for *p,p’*-DDE.

In Figures 2 and 3, we present the associations of DDT and DDE for these children at younger ages. We observed a significant trend with age at follow-up towards a positive association between DDT and DDE exposure and BMI *z*-score (*p-*interaction = 0.123 for *o,p*´-DDT; *p-*interaction = 0.087 for *p,p*´-DDT; *p-*interaction = 0.196 for *p,p*´-DDE). For odds of obesity (see Figure 3), we observed a significant trend with age at follow-up towards a positive association between *o,p*´-DDT (*p-*interaction = 0.192) and *p,p*´-DDE (*p-*interaction = 0.185) but not *p,p*´-DDT (*p-*interaction = 0.215) exposure. We maintained interaction terms in all models for consistency. When we limited the sample to children who had complete data at all four follow-up ages, the results were comparable (data not shown).

## Discussion

This longitudinal birth cohort study of a predominantly Mexican-American population residing in a California agricultural community provides some evidence that *in utero* DDT and DDE exposure may alter risk for obesity with age. We found that current data do not support a statistically significant positive association between *in utero* DDT and DDE exposure and obesity status of 7-year-old children. However, we observed a significant trend with age (2, 3.5, 5, 7 years) toward a positive association between maternal serum concentrations of DDT and DDE and odds of childhood obesity, providing support for further research in aging children.

The prevalence of obesity at 7 years of age in this study is very high (36%). This prevalence is higher than the 18% prevalence reported in NHANES for all U.S. children, 6–11 years old, and also higher than the 22% prevalence reported for Mexican-American children of the same age range (Ogden et al. 2012). Further, more than half of the children in this study were overweight or obese, and 1% had a BMI of ≥ 30 (the adult definition of obesity). In fact, the prevalence of overweight/obesity (53.3%) in this study is twice the prevalence (26.7%) reported in the Spanish prospective birth cohort study with a similar length of follow-up (6.5 years) (Valvi et al. 2012).

The results are consistent with the positive associations reported in the three prospective birth cohort studies that used a direct measure of prenatal DDT and DDE exposure (maternal serum at pregnancy or cord blood) and a standardized measure of overweight (BMI *z*-score ≥ 85th percentile) at 14 months of age (Mendez et al. 2011), at 3 years of age (Verhulst et al. 2009), and at 6.5 years of age (Valvi et al. 2012). Valvi et al. (2012) reported a nonmonotonic increase in the risk for overweight at 6.5 years that was modified by child sex; the risk with cord blood DDT exposure was limited to males. In contrast to Valvi et al. (2012), we found no difference in associations by child sex with either DDT or DDE exposure. Consistent with other previous studies (Mendez et al. 2011; Valvi et al. 2012; Verhulst et al. 2009), we found no change in associations after excluding low birth weight and preterm children. We also found no evidence of a relation of DDT or DDE exposure with waist circumference, but these children are still young and waist circumference may not be as sensitive a measure of overweight status in prepubertal populations. To our knowledge, no other studies have examined the relation of DDT and DDE exposure on waist circumference.

In this study, we found similar associations of *o,p*´-DDT and *p,p*´-DDT on 7-year-old child obesity status. Two other studies have examined prenatal *o,p*´-DDT and *p,p*´-DDT separately but neither used an age- and sex-standardized measure of BMI (Gladen et al. 2004; Jusko et al. 2006). Jusko et al. (2006) found no association between maternal serum *o,p*´-DDT, *p,p*´-DDT or *p,p*´*-*DDE and child weight *z*-score or height *z*-score at 5 years of age, but did not examine BMI *z*-score. In a subsample of 304 males from the Collaborative Perinatal Project, no association was reported between prenatal *o,p*´-DDT, *p,p*´-DDT or *p,p*´-DDE exposure and BMI with follow-up to 10–20 years of age (Gladen et al. 2004). Given the very wide age range of follow-up, interpretation is limited without an age-standardized measure of BMI (*z*-score). We found similar associations for *p,p*´-DDT or *p,p*´-DDE exposure on 7-year-old child obesity status measures. Given the high correlation in this study between maternal serum levels of DDT and DDE (*r* = 0.8 to 0.9), it is difficult to separate out the individual associations of each compound.

The results of this study are biologically plausible. In experimental studies, both DDT and DDE have been associated with adipose dysfunction (Howell and Mangum 2011; Moreno-Aliaga and Matsumura 2002). Given that DDT, an estrogen agonist, is metabolized to DDE, an androgen antagonist, there may be more than one mechanism to consider. Early developmental exposure to DDT and DDE could affect weight by affecting normal weight homeostasis either directly on adipose cells through differentiation and proliferation or indirectly via disruption of the endocrine feedback loop (Cooke and Naaz 2004; Diamanti-Kandarakis et al. 2009).

This study has several strengths. The CHAMACOS study is a longitudinal birth cohort with a relatively long follow-up period for which considerable information was collected about potential confounders. The study population is relatively homogenous (Mexican American) for factors such as diet, breastfeeding, country of origin, and socioeconomic status, which can reduce uncontrolled confounding. We were able to measure *o,p*´-DDT, *p,p*´-DDT, and *p,p*´-DDE exposure in maternal serum collected during the pregnancy. Exposure levels were high relative to other Mexican Americans (Centers for Disease Control and Prevention 2004), likely due to the mothers’ recent immigration from Mexico, but there was a wide range of exposure. Finally, we used a standardized measure of overweight based on BMI *z*-score which facilitates comparison across studies.

This study has some limitations. First, the positive associations we observed between *in utero* DDT and DDE exposure and obesity status at 7 years were not statistically significant; we cannot eliminate chance as an alternative explanation. It will be important to follow up the CHAMACOS cohort through puberty to examine the longitudinal trends in obesity with DDT and DDE exposure and to be able to consider the impact of such exposure on the adrenal hormone-mediated increase in weight and the sex steroid–induced pubertal growth spurt. Second, we were not able to consider the potential confounding effects of other chemical exposures such as polychlorinated biphenyls (PCBs) or hexachlorobenzene that have previously been associated with child obesity (Smink et al. 2008; Valvi et al. 2012). However, in CHAMACOS, concentrations of DDT and DDE were only weakly associated with PCB congeners and hexachlorobenzene (*r* = 0.09–0.19) (Chevrier et al. 2008). Finally, compared with the 417 mothers who had maternal serum DDT and DDE measurements, only 270 children had complete anthropometric data at 7 years. However, *in utero* DDT and DDE exposure levels of those with and without 7-year anthropometric data were not significantly different. In addition, predictors of maternal levels of *o,p*´-DDT, *p,p*´-DDT, and *p,p*´-DDE were similar to those reported previously in the larger group (Bradman et al. 2007).

In summary, we examined the association of *in utero* exposure to *o,p*´-DDT, *p,p*´-DDT, and *p,p*´-DDE with body weight in the CHAMACOS longitudinal birth cohort through 7 years of age. The present data do not support a statistically significant association between *in utero* DDT and DDE exposure and obesity status of 7-year-old children. However, the need for further research in aging children is supported by the statistically significant trend with age (2, 3.5, 5, 7 years) toward a positive association between maternal serum concentrations of DDT and DDE and the odds of childhood obesity. Continued follow-up of the CHAMACOS cohort will be informative.
